# Myosteatosis: A Distinct, Early and Targetable Novel Biomarker of Cancer Prognosis

**DOI:** 10.3390/cancers18060974

**Published:** 2026-03-18

**Authors:** Nagi B. Kumar

**Affiliations:** 1 Cancer Epidemiology Program, Moffitt Cancer Center and Research Institute, MRC-CAN CONT, 13131 Magnolia Drive, Tampa, FL 33612, USA; nagi.kumar@moffitt.org; Tel.: +1-813-745-6885; 2 Genitourinary Oncology Program, Moffitt Cancer Center and Research Institute, MRC-CAN CONT, 13131 Magnolia Drive, Tampa, FL 33612, USA; 3 Breast Oncology Program, Moffitt Cancer Center and Research Institute, MRC-CAN CONT, 13131 Magnolia Drive, Tampa, FL 33612, USA

**Keywords:** myosteatosis, cancer cachexia, sarcopenia, metabolic abnormalities, quantifying myosteatosis, cancer outcomes, cancer prognosis, treatment-related adverse effects

## Abstract

**Background:** Myosteatosis—fat infiltration within skeletal muscle—is emerging as a critical yet underappreciated determinant of cancer outcomes. Although historically overshadowed by sarcopenia and cachexia, myosteatosis is now recognized as a distinct and early muscle phenotype that carries significant prognostic implications across multiple tumor types. Despite its relevance, foundational gaps remain in our understanding of its mechanisms, clinical significance, and therapeutic potential. The goal of this manuscript is to review the current literature to identify the unique characteristics, prevalence, biological and other etiological mechanisms of myosteatosis and its role as an early biomarker in cancer treatment, recurrence, and prognosis. **Methods:** A review of the evidence from epidemiological, laboratory and other observational studies with regard to the definition of the biomarker, its biological mechanism, its prevalence in cancer patient populations and the gaps in the research was conducted. **Results:** Retrospective studies utilizing CT imaging reveal that myosteatosis is highly prevalent in cancer populations and independently associated with mortality, treatment toxicity, postoperative complications, functional decline, and survivorship outcomes. Unlike sarcopenia, which primarily reflects loss of muscle mass, myosteatosis reflects impaired muscle quality, metabolic dysfunction, and lipid derangements. Importantly, it appears early in the trajectory of muscle deterioration—well before irreversible cachexia develops—making it a strategic point for intervention. **Conclusions:** Despite strong associations with clinical outcomes, the biological underpinnings, clinical applications, and modifiability of myosteatosis remain poorly defined. A coordinated research agenda focused on mechanistic discovery, biomarker standardization, and targeted interventions is essential to improving survival, treatment tolerance, and quality of life for patients across the cancer continuum.

## Simple Summary

Myosteatosis is a condition where fat builds up inside and around muscles. Although not yet widely recognized, growing evidence shows it can strongly affect how people with cancer respond to treatment and how well they do over time. This paper reviews existing research to explain what myosteatosis is, how common it is, why it develops, and how it influences cancer treatment side effects, the chance of cancer coming back, and overall survival. Importantly, the review shows that myosteatosis may appear early, before obvious weight or muscle loss, making it a promising early warning sign of poor outcomes. The review also reveals major gaps in current knowledge. To move the field forward, coordinated research is needed to better understand the biology of myosteatosis, agree on how it should be measured, and develop targeted strategies to prevent or treat it. Addressing myosteatosis could help patients tolerate cancer treatments better, live longer, and maintain a better quality of life throughout their cancer journey.

## 1. Introduction

Globally, an estimated 20 million new cancer cases will be diagnosed in 2025, resulting in over 10.3 million deaths [World Health Association, 2025 [1]]. In the US, it is estimated that over 2 million new cancer cases will be diagnosed in 2025, and more than 618,000 people will die from the disease, the equivalent of about 1700 deaths per day [1]. Despite some progress with early detection and treatment of some cancers, globally, the numbers of cancer diagnoses and deaths are projected to rise substantially from 2024 to 2050 by 60.7% and 74.5%, respectively. This will result in an estimated 30.5 million new cancer cases and 18.6 million deaths by 2050 [2]. These findings clearly justify an urgent need to invest in meeting this challenge and continue to identify factors that can prevent and treat cancers and develop strategies to mitigate or eliminate both cancer- and treatment- induced adverse effects that impact overall survival and quality of life.

One of the most challenging adverse effects of cancer and cancer treatment that impacts prognosis is cancer cachexia (CC). CC is a pathological loss of striated muscle (skeletal and cardiac) and fat stores, manifesting in the cardinal features of emaciation, weakness affecting functional status, impaired immune system, metabolic dysfunction and poor quality of life [3]. CC occurs in 50% to 80% of cancer patients, based on varying definitions of this state. The etiology of CC is multifactorial and complex. The most clinically relevant phenotypic feature of CC is muscle loss (sarcopenia), resulting in [4] asthenia, fatigue, impaired physical function, reduced tolerance to treatments, increased toxicity to treatments, impaired quality of life and reduced survival. Sarcopenia, a hallmark of cancer cachexia, is present in 20–70% of patients, depending on the patient’s age, physical fitness, nutritional status, tumor type, grade of cancer at diagnosis, types of treatment and other comorbidities. Sarcopenia is classically defined as the age-related decline in skeletal muscle mass and, thus, function [5]. Sarcopenia is intrinsic to the ageing process but has been shown to be accelerated by various factors, including age-related inflammation (inflammaging), inactivity, poor nutrition, and chronic illness [6], including cancer. Sarcopenia is characterized by low skeletal muscle mass and reflects catabolic loss of muscle tissue. Studies have shown a correlation between sarcopenia and cancer treatment-related toxicity as well as overall prognosis [7,8,9]. Similar to CC, there are no acceptable diagnostic criteria [10], as reflected by the lack of a single clinical definition and the variety of measurement tools, measures and cutoff points used for diagnosis of sarcopenia [11]. Although much effort has been invested over the past two decades in defining and understanding the underlying mechanism and natural history of sarcopenia and CC, to date, CC and related sarcopenia continue to pose a critical challenge to the field of medical oncology, establishing the need to identify other early and targetable biomarkers in cancer patients. Once cancer cachexia is established, it is largely irreversible with conventional nutrition, exercise, or pharmacologic interventions.

Skeletal muscle abnormalities are highly prevalent in patients with cancer and strongly predict clinical outcomes; however, they represent distinct biological processes with different implications for intervention and prognosis. Skeletal muscle fat infiltration was originally observed to progressively increase with aging, contributing to frailty and metabolic syndrome (insulin resistance, diabetes). More recently, cancer-related MYO has been evaluated as an independent predictor of poor prognosis, independent of sarcopenia, in cancer patients undergoing treatment [12,13]. We hypothesize that MYO is distinct from sarcopenia and CC and may serve as a unique and novel biomarker that is more tangible for targeted interventions relative to sarcopenia or the complex syndrome of CC.

The goal of this manuscript is to review the current literature to define and identify the unique characteristics, prevalence, biological and other etiological mechanisms of myosteatosis (MYO) specific to cancer and its role as an early biomarker in cancer treatment and recurrence/prognosis.

## 2. Materials and Methods

This study was conducted as a structured narrative review aimed at synthesizing current evidence regarding the definition, biological mechanisms, prevalence, clinical implications, and research gaps related to myosteatosis in oncology. A narrative review approach was selected to integrate findings across heterogeneous study designs, including epidemiologic, imaging-based, translational, and clinical investigations, which are not readily amenable to formal meta-analysis. A literature search was performed using the following electronic databases: PubMed/MEDLINE, Embase, Scopus, and Web of Science. The search covered studies published from January 2000 through December 2025, reflecting the period during which imaging-based characterization of myosteatosis emerged in oncology research. Search terms included combinations of Medical Subject Headings (MeSH) and relevant keywords related to myosteatosis and cancer outcomes, including: “myosteatosis,” “muscle radiodensity,” “skeletal muscle attenuation,” “intramuscular fat,” and “muscle quality,” combined with “cancer,” “oncology,” “survival,” “treatment toxicity,” “cachexia,” “sarcopenia,” “body composition,” “computed tomography,” and “CT imaging.” Reference lists of relevant reviews and primary articles were also manually screened to identify additional eligible studies.

Studies were included if they evaluated myosteatosis or muscle radiodensity/intramuscular adiposity using imaging or validated assessment methods, included adult cancer populations, examined associations with clinical, functional, metabolic, or survival outcomes, and provided mechanistic or biological insights relevant to myosteatosis. The review included observational cohort studies, retrospective imaging analyses, translational studies, and relevant mechanistic investigations. Studies were excluded if they focused exclusively on non-cancer populations without mechanistic relevance, examined sarcopenia without assessment of muscle quality or fat infiltration, were conference abstracts lacking sufficient methodological detail, or were non-English publications.

Study Selection and Data Synthesis: Titles and abstracts were screened for relevance, followed by full-text review of eligible articles. Evidence was synthesized qualitatively, with emphasis on recurring findings across tumor types, methodological approaches, and biological pathways. Given the heterogeneity in study design and outcome reporting, quantitative meta-analysis was not performed.

Assessment of Evidence Quality: Because this review was narrative in nature, formal risk-of-bias scoring was not conducted. However, interpretation of the evidence considered factors including sample size, methodological rigor, imaging standardization, adjustment for confounding variables, and consistency of findings across independent cohorts. A review of evidence from epidemiologic, laboratory, and observational studies addressing the definition of myosteatosis as a biomarker, its biological mechanisms, prevalence in cancer populations, and current research gaps was conducted. Because the objective of this review was conceptual synthesis rather than systematic evidence aggregation, a CONSORT flow diagram was not generated.

## 3. Results

**Figure 1:**
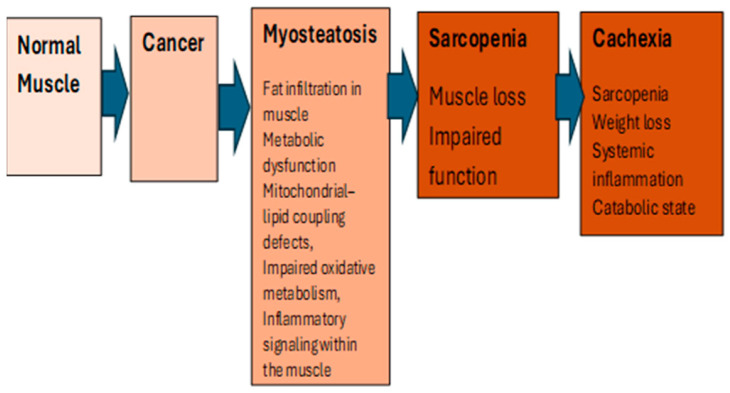
Distinction between normal muscle, myosteatosis, sarcopenia and cachexia in cancer patients.

MYO has been defined as excessive fat deposition (=steatosis) in skeletal muscle (=myo) [14] and can be identified as a relatively early biomarker of muscle quality, characterized by the excess deposition of fat within or between skeletal muscle fibers, which disrupts contractility and diminishes muscle function [15,16]. MYO has also been defined as ectopic infiltration of fat concurrently into visceral and skeletal muscles. In a non-small cell (NSC) lung cancer patient cohort, MYO was characterized as both low skeletal muscle radiodensity (SMD) and high intramuscular adipose tissue (IMAT) [17]. MYO is increasingly recognized as critical to muscle mass, strength, quality and function. Although MYO is often considered synonymous with sarcopenia, it is a unique syndrome and distinct from sarcopenia, and is marked by fatty infiltration into muscle, which induces proinflammatory changes that contribute to decreased muscle function, compromise mitochondrial function, and increase inflammatory response in muscles [18]. MYO may precede sarcopenia and cancer cachexia (Figure 1) and may be independent of muscle mass [7,19]. MYO is thus biologically and clinically distinct from sarcopenia, which reflects only reduced muscle mass, and from cancer cachexia, an advanced, largely irreversible catabolic state.

### 3.1. Biological Mechanisms

**Figure 2:**
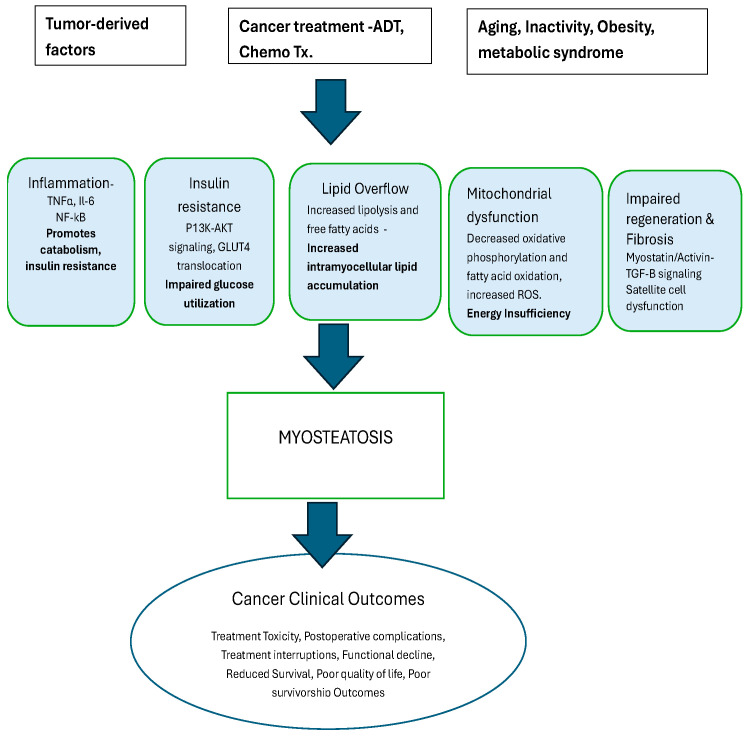
Biological mechanism underlying development and clinical consequences of myosteatosis in cancer.

Several investigators have attempted to evaluate the underlying mechanisms by which MYO occurs in cancer patients (Figure 2). These studies have shown that MYO arises from a convergence of metabolic, inflammatory, endocrine, and cellular disruptions that impair muscle quality and promote intramuscular fat accumulation. MYO has been correlated with an upregulation in the secretion of pro-inflammatory factors, insulin resistance, and impaired immune function targeting various signaling pathways to increase tumor cell proliferation [20,21], with implications in cancer progression. In a preclinical model, Cardaci et al. examined skeletal muscle lipid abnormalities and lipid droplet (LD)–mitochondrial interactions in the Lewis lung carcinoma (LLC) mouse model of cancer cachexia. LLC-bearing mice showed a marked increase in intramyocellular lipid droplets—both in number and size—along with more oil red O-positive fibers and an inverse relationship between LD accumulation and myofiber size. LDs were more elongated and structurally abnormal, and LD–mitochondrial contacts were significantly reduced. Cachectic muscles also displayed altered expressions of lipid-handling proteins. Overall, LLC mice exhibited pronounced MYO, disrupted LD morphology, and impaired LD–mitochondrial coupling [22].

Others have reported progenitor cell/microenvironment changes with CC altering the muscle niche (NF-κB activation, cytokines, ECM remodeling), leading to expansion and adipogenic shift in FAP-like cells, producing the intermuscular adipose tissue seen in MYO [23]. Both muscle and visceral fat contribute to inflammatory pathways in this cascade. Experimental evidence has demonstrated that the ubiquitin–proteasome pathway, as indicated by upregulation of NF-kB, accounts for the majority of skeletal muscle degradation, stimulated by several proinflammatory cytokines including TNFα and Il-6 [22,24]. In a study aimed at exploring the association between systemic inflammation and MYO upon diagnosis of gastric cancers (GC), specially whether the co-occurrence of these factors could predict survival outcome, the team observed that the co-occurrence of MYO and inflammation increased disease progression and death risk by almost three times [25].

The accumulation of intramyocellular lipids—particularly diacylglycerols (DAGs) and ceramides—impairs insulin signaling within muscle fibers, leading to reduced activation of anabolic pathways and diminished muscle protein synthesis [26]. These lipid intermediates disrupt downstream insulin receptor signaling (e.g., IRS-1/AKT), producing a state of anabolic resistance that undermines normal muscle maintenance. In cancer, this process is further exacerbated by systemic inflammation, tumor-driven metabolic alterations, and catabolic cytokine signaling, all of which intensify lipid accumulation and compound insulin resistance [26,27]. As a result, insulin-related metabolic dysfunction becomes a central contributor to impaired muscle quality and the development of MYO in cancer patients.

Hormonal and systemic endocrine disturbances play a critical role in the development of MYO. Alterations in glucocorticoids, sex steroids, insulin, and adipokine signaling all influence muscle lipid deposition and metabolic dysfunction. Elevated glucocorticoid levels—whether endogenous (e.g., stress, illness) or exogenous (e.g., therapeutic steroids)—promote adipogenic differentiation of fibro-adipogenic progenitors (FAPs) within skeletal muscle, increasing intramuscular fat accumulation [26]. Similarly, sex-steroid deficiency, including estrogen loss in women and androgen suppression in men, contributes to reduced muscle anabolism, impaired mitochondrial function, and increased susceptibility to lipid infiltration [27,28]. Dysregulation of leptin, insulin, and adipokines further disrupts muscle–fat crosstalk, exacerbating metabolic inflexibility and promoting lipid storage within muscle fibers. In cancer, these endocrine abnormalities are amplified by disease-related metabolic stress and by specific treatments—such as androgen deprivation therapy (ADT) in prostate cancer—that accelerate muscle quality decline and may substantially enhance MYO [29].

Together, these pathways create a metabolic environment in which muscle fibers lose oxidative flexibility, accumulate lipids, and undergo structural and functional decline—positioning MYO as a distinct, early, and clinically meaningful marker of muscle dysfunction and poor cancer prognosis.

### 3.2. Quantifying MYO

To date, there is no single measure to quantify MYO that has been identified and validated in well-powered studies that can be applied clinically. Although muscle biopsy remains the gold standard for detecting adipose infiltration in muscle, clinical assessment is invasive and impractical. Additionally, the quantification in whole body may not be possible to achieve. Thus, clinical assessment using this technique may be mostly used in research setting for localized quantification. Thus, the quantification of MYO is currently performed using imaging techniques such as data from PET scans of the whole body, or utilizing images from computed tomography (CT) or magnetic resonance imaging (MRI) obtained for cancer diagnosis or follow-up to evaluate muscle attenuation in selected cross-sectional areas [30,31]. However, due to varying definitions of MYO, specifically the inclusion of skeletal and/or visceral muscles, differences in methodologies to assess fat infiltration and function, as well as in the applied references values for the extent of MYO, there is no universal consensus. The most widely used modality to diagnose MYO is abdominal CT, due to its routine acquisition during cancer staging and surveillance, based on evaluation of the muscle radiodensity of the total abdominal muscle area, predominantly at the L3 vertebral level [32,33]. Skeletal muscle radiodensity (SMD), measured in Hounsfield Units (HU), serves as a surrogate marker of lipid infiltration within muscle tissue. Over the past two decades, the third lumbar vertebra (L3) has emerged as the preferred anatomical landmark because cross-sectional muscle area and composition at this level strongly correlate with whole-body skeletal muscle mass and adiposity. L3 includes multiple major muscle groups (psoas, paraspinal, and abdominal wall muscles), providing a representative assessment of systemic muscle composition. In a meta-analysis to identify studies that used CT muscle measurements to assess muscle mass and myosteatosis, skeletal muscle index and intermuscular adipose tissue were the most commonly used measures of abdominal wall muscle mass (114/142) and myosteatosis (27/49), respectively. However, cutoff points varied across studies, with a majority of studies failing to report important technical CT parameters, such as the use of intravenous contrast and slice thickness (94% and 63%, respectively) [34]. Although often used interchangeably, SMD and IMAT capture distinct aspects of muscle adiposity. Skeletal Muscle Radiodensity (SMD) reflects diffuse lipid infiltration within muscle fibers and extracellular spaces, inferred from reduced attenuation values on CT. SMD is thought to capture metabolic dysfunction, mitochondrial impairment, and intracellular lipid accumulation. Intermuscular Adipose Tissue (IMAT), on the other hand, represents discrete fat depots located between muscle groups beneath the fascia, quantified as adipose tissue area rather than attenuation. Emerging evidence suggests that reduced SMD may be more strongly associated with systemic metabolic dysregulation and treatment toxicity, whereas IMAT may reflect structural adiposity and physical function impairment. These phenotypes likely represent complementary but biologically non-identical processes within the spectrum of myosteatosis [35]. Others have utilized a single CT image at the L4 pedicle level, including measurements of visceral adipose tissue (VAT), subcutaneous adipose tissue (SAT), and skeletal muscle area, with attenuation obtained using clinical PACS and specialized segmentation software. These changes have been used to predict clinical overall survival in cancer patient populations [36]. Based on the specific cancer, alternative vertebral levels that are routinely captured via CT present significant limitations. For example, cervical (C3), available in head and neck cancer patients, may be less representative of total-body muscle composition; smaller muscle groups increase variability. Similarly, thoracic (T12), often included in chest CT, provides partial muscle representation but has respiratory motion artifacts. Additionally, contrast administration can influence attenuation values by increasing measured HU, potentially affecting SMD thresholds. These small changes can contribute to misclassification issues when fixed cutoff values are used to diagnose myosteatosis with CT, underscoring the importance of reporting absolute values and the specific contrast phase used in future studies. Others have reported similar findings and recommendations [37,38]. Thus, a failure to account for contrast status represents a potential source of measurement heterogeneity across studies. Based on these early observations, it is clear that although CT at L3 is currently used in most research and clinical settings to quantify MYO, this methodology is fraught with significant heterogeneity. Studies reporting retrospective data have utilized differing cutoff values to diagnose MYO (32 different cutoff values among 73 studies) [39]. Additionally, the current imaging measures fail to combine metabolic abnormalities or other mechanism-based biomarkers that may more comprehensively define this phenotype.

Prevalence of Myosteatosis in Cancer Patient Populations:

The reported prevalence of myosteatosis (MYO) among cancer populations varies widely, generally ranging from approximately 30% to 60%, and in some cohorts exceeding these estimates depending on tumor type, disease stage, treatment exposure, comorbid conditions, imaging modality, and the diagnostic thresholds used to define reduced muscle attenuation or increased intramuscular adipose tissue [17,40]. Variability in prevalence estimates is largely attributable to differences in imaging protocols, anatomical measurement sites, and cutoff points applied to skeletal muscle radiodensity.

Studies across gastrointestinal malignancies demonstrate particularly high prevalence rates. In patients with esophagogastric cancer, MYO prevalence has been reported to be as high as 87.6% [41]. Similarly, in a smaller cohort of individuals with esophageal, gastric, or pancreatic cancers, approximately 60% met the criteria for MYO using CT-derived muscle density parameters [42]. Among pancreatic and periampullary adenocarcinoma patients, MYO was identified in 25% of individuals, with an additional subset exhibiting concurrent sarcopenia, highlighting the overlapping yet distinct occurrence of muscle quality and quantity abnormalities [43].

In colorectal cancer (CRC), MYO is frequently observed when assessed using CT imaging at the third lumbar vertebra (L3), although reported prevalence varies depending on population characteristics and diagnostic thresholds [13,14,44]. Similarly, MYO is commonly identified in lung cancer populations. Analyses of non-small cell lung cancer (NSCLC) cohorts using both abdominal and thoracic CT imaging demonstrate substantial prevalence of reduced skeletal muscle radiodensity and increased intramuscular adipose tissue [17,35].

Across studies, differences in prevalence estimates reflect methodological heterogeneity, including retrospective study designs, variation in imaging acquisition (contrast vs. non-contrast CT), inconsistent anatomical landmarks, and a lack of standardized consensus cutoff points for MYO classification. Despite these limitations, available evidence consistently indicates that MYO is highly prevalent across multiple solid tumor types and disease stages, underscoring the need for standardized diagnostic criteria and harmonized reporting methods to enable cross-study comparisons. While the prevalence of myosteatosis across diverse cancer populations is now increasingly recognized, comprehensively defining MYO and understanding its clinical implications—including treatment tolerance, postoperative complications, and survival outcomes—remains essential to determining whether MYO represents merely a descriptive imaging phenotype or a clinically actionable biomarker.

## 4. Discussion

Although historically overshadowed by sarcopenia and cancer cachexia, myosteatosis (MYO) is increasingly recognized as a distinct and early muscle phenotype with important prognostic implications across multiple tumor types. Despite its growing relevance, substantial foundational gaps remain in our understanding of its biological mechanisms, clinical significance, and therapeutic potential. The cellular and molecular drivers of MYO in cancer, including mitochondrial dysfunction, altered lipid droplet biology, inflammatory signaling, fibro-adipogenic progenitor fate shifts, and systemic endocrine perturbations, have not been systematically investigated in well-characterized human cancer populations.

Table 1 summarizes representative clinical studies evaluating myosteatosis across major tumor types, highlighting common imaging approaches, measurement methods, and associated clinical outcomes. In all these studies, evaluating various stages of cancers and cancers ranging from colorectal to hematologic tumors [34,45,46,47,48,49,50,51,52,53,54,55,56,57,58,59,60,61], MYO was defined merely utilizing the imaging modality of CT at L3 region. The data in summary clearly indicate that MYO impacts response to treatment, increases treatment toxicity, treatment interruptions, infection risk, and mortality, and decreases functional status, quality of life and overall survival. Accumulating evidence indicates that myosteatosis reflects interconnected alterations in skeletal muscle biology, metabolic regulation, and immune signaling that collectively influence cancer prognosis and treatment outcomes. However, the field remains limited by the absence of standardized definitions and imaging methodologies for MYO quantification. Variability in CT attenuation thresholds, anatomical assessment sites, acquisition parameters, and interpretative approaches restricts comparability across studies and hinders clinical adoption. Furthermore, most available evidence derives from retrospective cohorts. Although metabolic, immune, and endocrine alterations associated with MYO have been independently documented, their integrated and concurrent effects have not been comprehensively characterized.

The natural history of MYO within specific cancer populations remains largely unknown. Critical gaps include the timing of onset, rate of progression during treatment, and potential reversibility of early-stage changes. Prospective longitudinal studies defining the temporal trajectories of MYO, alongside rigorously collected biomarker validation cohorts, are urgently needed. Identification of composite biomarker panels integrating imaging-derived muscle quality with metabolic and immune indicators may be essential, as body composition measures alone fail to capture the full spectrum of risk. Importantly, MYO remains understudied in key clinical populations, including prostate cancer patients receiving androgen deprivation therapy, breast cancer patients undergoing endocrine therapies, early-stage survivors, racially and ethnically diverse populations, and metabolically vulnerable subgroups.

Future targeted intervention trials should determine whether MYO represents a modifiable phenotype. Strategies incorporating precision nutrition, structured exercise, metabolic therapies, and endocrine modulation must evaluate whether reversal or attenuation of MYO improves treatment tolerance, survival outcomes, and patient-reported quality of life. Standardization of imaging definitions, integration of metabolic and immune biomarkers, and both retrospective and prospective longitudinal validation studies represent essential next steps toward clinical implementation. By integrating imaging phenotypes with underlying biological mechanisms, myosteatosis could emerge not merely as a marker of disease burden but also as a potentially modifiable target, providing a framework for precision risk stratification and intervention across the cancer continuum.

## 5. Conclusions

In conclusion, accumulating evidence indicates that myosteatosis (MYO) represents a powerful yet under-recognized biomarker of adverse prognosis in cancer, reflecting early alterations in skeletal muscle quality, metabolic dysregulation, and inflammatory signaling. Its conceptual and biological distinction from sarcopenia and cancer cachexia highlights MYO as a unique and potentially targetable muscle phenotype with important implications for cancer outcomes. As imaging-based body composition analysis becomes increasingly integrated into routine oncologic care, standardized assessment of MYO may offer an opportunity to identify high-risk patients earlier in the disease trajectory, enabling interventions aimed at improving treatment tolerance, reducing toxicity, and preserving functional status. A coordinated research agenda focused on mechanistic discovery, biomarker standardization, and targeted intervention strategies will be essential to advance the science of MYO. By addressing these gaps, the field has the opportunity to transform MYO from an overlooked imaging observation into a clinically actionable determinant of cancer prognosis, ultimately improving survival, treatment tolerance, and quality of life for patients across the cancer continuum. Future prospective studies and clinical trials incorporating standardized MYO assessment will be critical to determine whether targeting muscle quality can meaningfully improve oncologic outcomes.

## Figures and Tables

**Table 1:** Clinical studies evaluating myosteatosis across cancer types: measurement approaches and associated outcomes.

Tumor Type	Study Population	Imaging Modality	Myosteatosis Measure	Clinical Outcomes	References
Colorectal cancer	Resected and metastatic CRC	CT L3	Skeletal Muscle Radiodensity (SMD, HU)	↓ overall survival; ↑ postoperative complications; ↑ chemotherapy toxicity	[45,46]
Pancreatic cancer	Surgical resection cohorts	CT L3	SMD; IMAT	↑ mortality; ↑ length of stay; ↑ surgical morbidity	[47,48]
Hepatocellular carcinoma	Cirrhosis + HCC	CT L3	SMD	↓ survival; ↑ liver decompensation	[49,50]
Lung cancer (NSCLC)	Chemoradiation and advanced disease	CT L3/T12	SMD	↓ survival; ↑ treatment toxicity; ↓ functional status	[51,52]
Breast cancer	Early and metastatic	CT opportunistic	SMD	↑ treatment toxicity; ↓ survival (selected cohorts)	[53,54]
Prostate cancer	ADT-treated patients	CT L3; MRI thigh	SMD; MRI fat fraction	metabolic dysfunction; functional decline; adverse body composition	[55,56]
Head and neck cancer	Definitive CRT	CT C3 (converted to L3)	SMD	↓ survival; ↑ treatment interruptions	[57,58]
Hematologic malignancies	Lymphoma/transplant	CT L3	SMD	↑ infection risk; ↓ survival	[59]
Mixed solid tumors	Pan-cancer cohorts	CT L3	SMD ± IMAT	↑ mortality; ↑ toxicity; ↓ QoL	[60,61]

Table Abbreviations: CT, computed tomography; MRI, magnetic resonance imaging; L3, third lumbar vertebra; C3, third cervical vertebra; T12, twelfth thoracic vertebra; SMD, skeletal muscle radiodensity; IMAT, intermuscular adipose tissue; HU, Hounsfield units; ADT, androgen deprivation therapy; CRT, chemoradiotherapy.

## Data Availability

Not applicable.

## Abbreviations

The following abbreviations are used in this manuscript:

MYO	Myosteatosis
CC	Cancer cachexia
NSC	Non-small cell
SMD	Skeletal muscle radiodensity
IMAT	Intramuscular adipose tissue
LLC	Lewis lung carcinoma
LD	Low density
NF-κB	Nuclear factor kappa-light-chain-enhancer of activated B cells
TNFα	Tumor necrosis factor-alpha
Il	Interleukins
GC	Gastric cancer
DAGs	Diacylglycerols
IRS	Insulin receptor signaling
AKT	Protein kinase
CD3+/CD4+ cells	Cluster of differentiation 3 and 4 cells
AMG	Albumin MYO gauge
CT	Computed tomography
L3	Lumbar vertebrae 3
SMI	Skeletal muscle index (SMI)
CRC	Colorectal cancer
ADT	Androgen deprivation therapy
OS	Overall survival
CSS	Cancer-specific survival
DFS	Disease-free survival
